# Biclonal Gammopathy as a Misleading Indicator to Diagnose POEMS Syndrome: An Autopsy Case Report and a Review of the Literature

**DOI:** 10.7759/cureus.25153

**Published:** 2022-05-20

**Authors:** Shizuko Ainai, Ryouhei Komaki, Naokazu Muramae, Rena Uno, Kenta Mori, Kazunori Otsui, Kimikazu Yakushijin, Kazuhiko Sakaguchi

**Affiliations:** 1 Department of Rheumatology and Clinical Immunology, Kobe University Hospital, Kobe, JPN; 2 Division of Neurology, Kobe University Hospital, Kobe, JPN; 3 Department of General Internal Medicine, Kobe University Graduate School of Medicine, Kobe, JPN; 4 Department of Diagnostic Pathology, Kobe University Hospital, Kobe, JPN; 5 Department of General Internal Medicine, Kobe University Hospital, Kobe, JPN; 6 Department of Medical Oncology, Kobe University Hospital, Kobe, JPN

**Keywords:** plasma cell myeloma, discrepancy, clonality, biclonal gammopathy, poems syndrome

## Abstract

A 76-year-old man presented with a four-month history of progressive bilateral lower limb muscle weakness and dysesthesia. The patient had extravascular volume overload, and laboratory findings confirmed hypothyroidism, renal dysfunction, and chronic inflammation. Serum protein and immunofixation electrophoresis revealed biclonality of immunoglobulin A (IgA)-kappa and IgA-lambda, which was attributed to chronic inflammation. Subsequently, we detected the proliferation of monoclonal plasma cells in the bone marrow, which led to a diagnosis of POEMS syndrome. Despite the initiation of chemotherapy, the patient died of aspiration pneumonia. In this case, biclonal gammopathy in peripheral blood delayed a diagnosis of POEMS syndrome.

## Introduction

POEMS syndrome is a paraneoplastic disorder associated with the proliferation of plasma cell-derived cells and characterized by polyneuropathy, organomegaly, endocrinopathy, M-protein, and skin lesions [1]. Monoclonal gammopathy is commonly seen in patients with POEMS syndrome and comprises monoclonal immunoglobulin (Ig)G/IgA-lambda [2]. Monoclonality is a specific characteristic of this syndrome, although there are some reports of affected individuals with biclonal gammopathy in peripheral blood or urine samples. However, most cases do not include a complete immunohistopathological examination of bone marrow or other plasmacytoma, which are sites of plasma cell proliferation [3,4]. Although there are a few reports of biclonal gammopathies with lambda-restricted plasma cells in localized plasmacytoma and with kappa-restricted plasma cells in bone marrow [3], in other cases, serum protein electrophoresis (SPEP) has confirmed biclonality in serum only [5-11].

Biclonality makes the correct and prompt diagnosis of POEMS syndrome challenging, especially in cases that do not show M-protein in peripheral blood and urine. In this report, we describe a patient with POEMS syndrome in whom SPEP and immunofixation electrophoresis (IFE) suggested biclonality of IgA-kappa and IgA-lambda but was later found to have a proliferation of lambda-restricted monoclonal plasma cells in the bone marrow. The disease progressed rapidly in this patient with overproduction of pro-inflammatory cytokines and renal dysfunction.

## Case presentation

The patient was a 76-year-old man who had been well until four months prior to admission when he started to have difficulty in raising both lower limbs more than 30 cm and climbing stairs. Three months ago, he lost strength in his ankles. At the same time, he became aware of dysesthesia on the soles of both feet. Although sensory abnormalities remained at the same level, the weakness of the lower extremities gradually progressed and was accompanied by edema. This resulted in admission to another hospital one month earlier. He was initially diagnosed with pericarditis because of pericardial and pleural effusion and was treated with aspirin 2.4 g/day and colchicine 0.5 mg/day. However, the muscle weakness in his lower limbs gradually worsened to the point that he could not walk without assistance, and he was transferred to our hospital.

His medical history was unremarkable except for lumbar spondylolisthesis, cholecystectomy, and hypertension. On physical examination, his height and body weight were 162 cm and 56.0 kg, respectively. An ejection systolic murmur was heard (Levine III/VI, maximum at the apex). He had hyperpigmentation extending from the right lateral area of the abdomen to the back, although the photograph had not remained. There was no hepatosplenomegaly, ascites, or hypertrichosis. Slow pitting lower limb edema and right-sided inguinal lymphadenopathy were present. He had diffuse muscle weakness in both lower limbs with areflexia and sensory disturbance, including dysesthesia, paresthesia, and decreased light touch sensation, on the soles of the feet. No osteolytic or osteoclastic lesions were evident on both systemic computed tomography (CT) and magnetic resonance imaging (MRI). The laboratory data on admission are summarized in Table 1.

**Table 1:** Laboratory data on admission.

Test	Result	Units	Range
White blood cells	9,900	/μL	3,300–8,600
Segmented neutrophil	72	%	38–58
Eosinophil	3	%	0–5
Basophil	1		0–1
Monocyte	5	%	2–8
Lymphocyte	19	%	26–47
Red blood cells	381	×10^4^/μL	386–492
Hemoglobin	11.2	g/dL	11.6–14.8
Platelet	50.0	×10^4^/μL	15.8–34.8
Total protein	6.5	g/dL	6.6–8.1
Albumin	3.3	g/dL	4.1–5.1
Aspartate aminotransferase	24	IU/L	13–30
Alanine aminotransferase	16	IU/L	7–23
Gamma-glutamyl transferase	25	IU/L	9–32
Lactate dehydrogenase	108	IU/L	124–222
Total bilirubin	100.1	mg/dL	0.4–1.5
Blood urea nitrogen	16.7	mg/dL	8–20
Creatinine	3.83	mg/dL	0.46–0.79
Estimated glomerular filtration rate	12.9	mL/minute/1.73 m^2^	60 -
Sodium	138	mmol/L	137–147
Potassium	5.0	mmol/L	3.5–5.0
Chlorine	110	mmol/L	98–108
Calcium	8.0	mg/dL	8.4–10.4
Phosphorus	6.9	mg/dL	2.6–4.6
Uric acid	16.0	mg/dL	3.6–6.9
Glucose	97	mg/dL	73–109
Thyroid-stimulating hormone	5.289	μIU/mL	0.5–5.5
Free thyroxine	0.44	ng/dL	1.1–1.8
Brain natriuretic peptide	40.93	pg/mL	- 18.4
C-reactive protein	4.26	mg/dL	-0.14
Soluble interleukin-2 receptor	493	U/mL	36.9–121.0
Immunoglobulin G	901	mg/dL	870–1,700
Immunoglobulin A	804	mg/dL	110–410
Immunoglobulin M	51	mg/dL	33–190
Vascular endothelial growth factor (serum)	7,380	pg/mL	143–659
Free light chain-kappa	179	mg/L	3.3–19.4
Free light chain-lambda	104	mg/L	5.7–26.3
Free light chain ratio	1.721		0.26–1.65
Interleukin-6	20.2	pg/mL	- 4.0
Tumor necrosis factor-alpha	7.84	pg/mL	0.6–2.8

**Figure 1:**
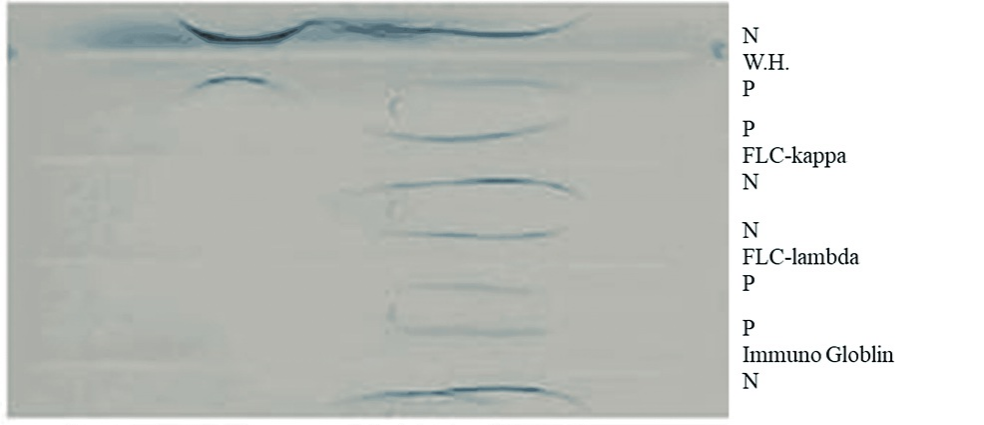
Urine protein electrophoresis. FLC-kappa: free light chain kappa; FLC-lambda: free light chain lambda; N: negative control; P: patient; WH: human whole serum

Urine protein electrophoresis (UPEP) suggested IgA-kappa gammopathy (Figure 1).

**Figure 2:**
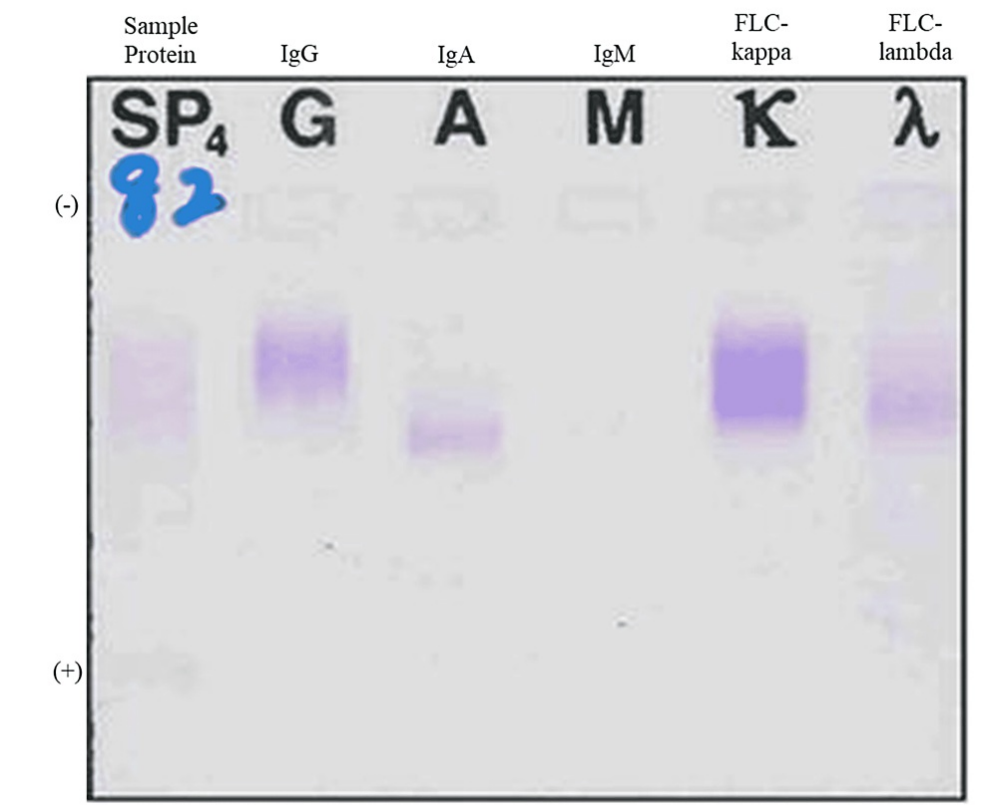
Immunofixation of urine protein. Immunofixation of urine confirmed FLC-kappa (black arrow). FLC-kappa: free light chain kappa; FLC-lambda: free light chain lambda; Ig: immunoglobulin

IFE of urine showed a trace amount of Bence-Jones protein kappa (Figure 2). The serum kappa free light chain (FLC-kappa) level was 179 mg/L (normal range: 3.3-19.4 mg/L) and the serum lambda free light chain (FLC-lambda) was 104 mg/L (normal range: 5.7-26.3 mg/L) [12]. The serum-free light chain ratio (FLCR) was within normal range.

**Figure 3:**
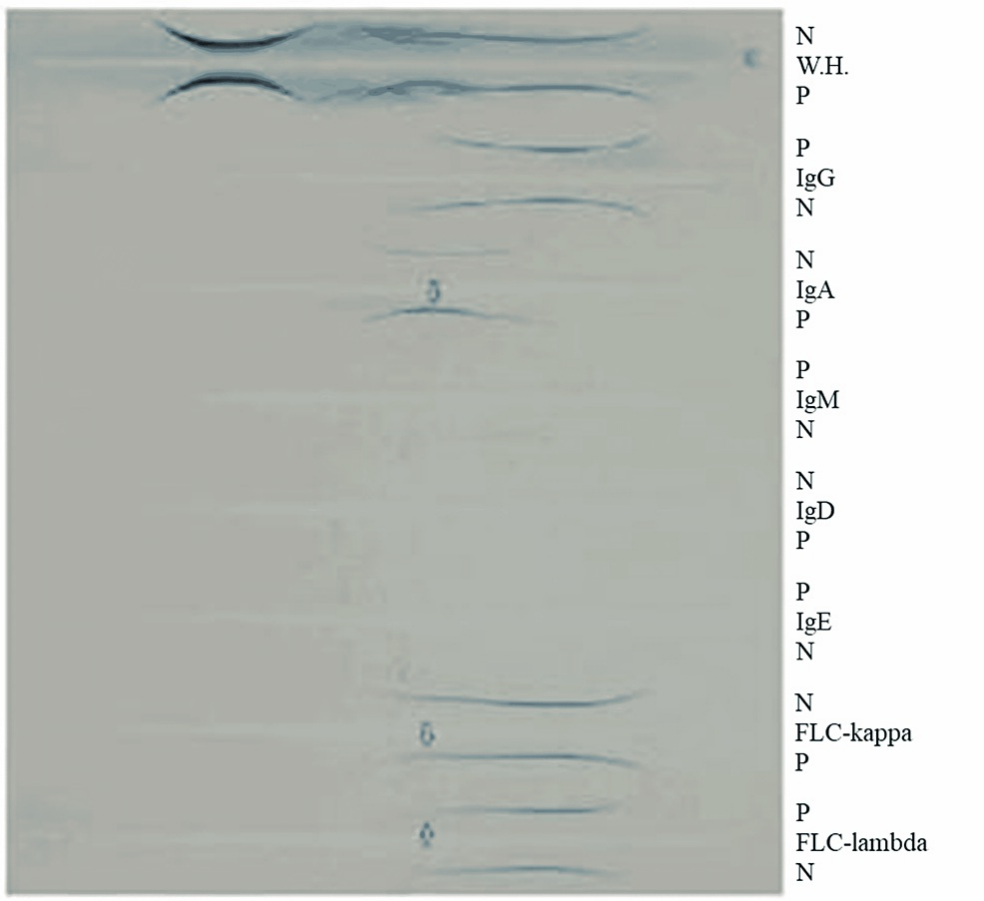
Electrophoresis of serum protein. FLC-kappa: free light chain kappa; FLC-lambda: free light chain lambda; N: negative control; P: patient; WH: human whole serum; Ig: immunoglobulin

Serum SPEP suggested biclonal gammopathy of IgA-kappa and IgA-lambda but in trace amounts (Figure 3).

**Figure 4:**
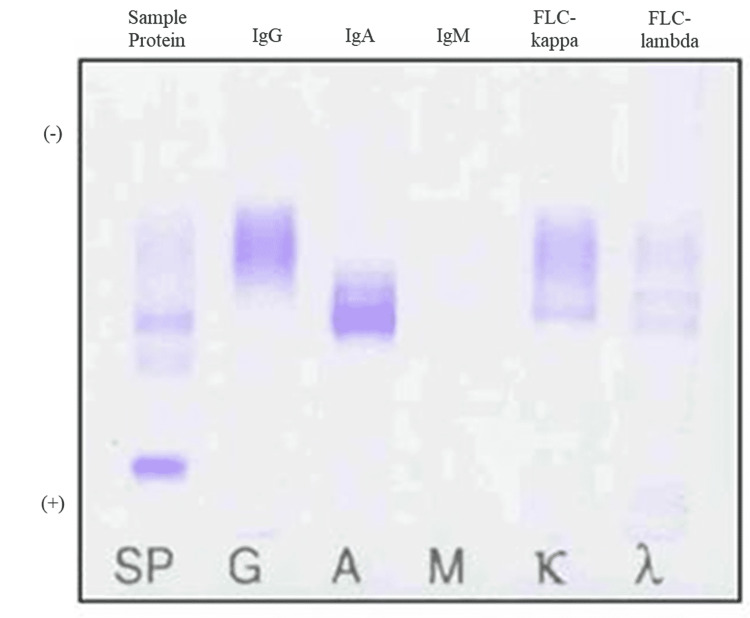
Immunofixation of serum protein. Serum immunofixation suggested IgA-kappa (black arrow) and IgA-lambda (white arrow) biclonal gammopathy. FLC-kappa: free light chain kappa; FLC-lambda: free light chain lambda; Ig: immunoglobulin

Serum IFE showed weak bands of both IgA-kappa and IgA-lambda on a background of polyclonal gammopathy (Figure 4).

**Figure 5:**
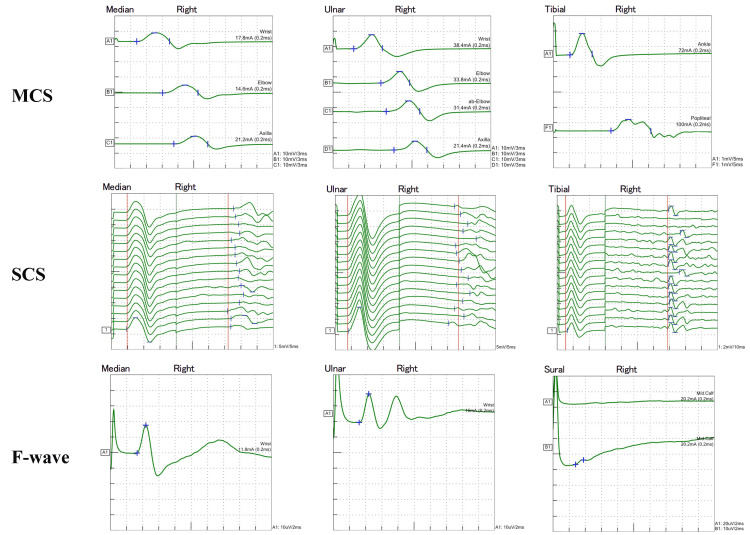
Nerve conduction study at the right upper and lower extremities on day one of admission. In MSC, prolonged DL, decreased MCV, and decreased CMAP were observed in all examined nerves. In SCS, the amplitude of SNAP was conserved in the median and ulnar nerves and reduced in the sural nerve. The SCV was decreased in all examined nerves. In F-wave, the maximal latency was prolonged in all examined nerves. These findings were compatible with motor predominant-sensorimotor demyelinating neuropathy. MSC: motor nerve conduction study; DL: distal latency; MCV: motor nerve conduction velocity; CMAP: compound muscle action potential; SCS: sensory nerve conduction study; SNAP: sensory nerve action potential; SNAP: sensory nerve action potential; SCV: sensory nerve conduction velocity

The serum vascular endothelial growth factor level was 7,380 pg/mL (cut-off value for diagnosis of POEMS syndrome: 1,920 pg/mL [1]). The tumor necrosis factor-alpha (TNF-α) level was elevated (20.2 pg/L; normal range: 2.27-11.2 pg/mL), as was the interleukin (IL)-6 level (7.84 pg/mL; normal range: <2.41 pg/mL). Nerve conduction studies performed on day one of admission revealed sensorimotor demyelinating neuropathy (Figure 5).

A CT scan on day five showed bilateral pleural effusion, pericardial effusion, and right-sided inguinal lymphadenopathy. An inguinal lymph node biopsy showed accumulation of lymphocytes and enlargement of endothelial cells, suggesting chronic inflammation. Bone marrow examination revealed an increase in abnormal plasma cells of 13%. There was a clustering proliferation of lambda monoclonal plasma cells on a background of diffuse proliferation of kappa plasma cells and an abnormal lambda/kappa FLCR of 10 to 1. These findings established the diagnosis of POEMS syndrome with plasma cell myeloma, even though there was no obvious monoclonality in serum or urine samples [1]. During these examinations, he required frequent abdominal paracentesis for severe ascites. Bortezomib and betamethasone were started on day 43 when plasma cell myeloma was pathologically confirmed. Unfortunately, there was no improvement, and the chemotherapy was stopped when the patient sustained a brain infarction. On day 78, he succumbed to aspiration pneumonia, after which an autopsy was performed.

**Figure 6:**
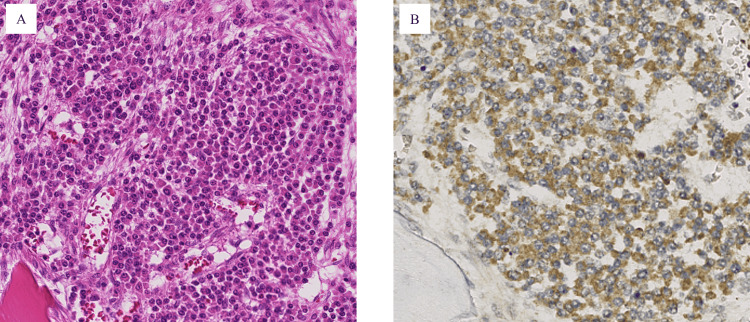
Vertebral bone marrow. (A) Patchy cluster of plasma cells (hematoxylin-eosin staining; original magnification 200×). (B) CD138-positive cells showing a predominance of lambda-chain (lambda immunohistochemical staining; original magnification 200×).

On gross examination, we observed chronic or subacute cerebral infarctions, bronchopneumonia, and some age-related lesions, which were confirmed histologically. The vertebral bone marrow contained patchy clusters of plasma cells (Figure 6A), which were positive for CD138, reported as a marker of the plasma cell [13], and showed a predominance of lambda chain (Figure 6B), thereby meeting the criteria for POEMS syndrome.

**Figure 7:**
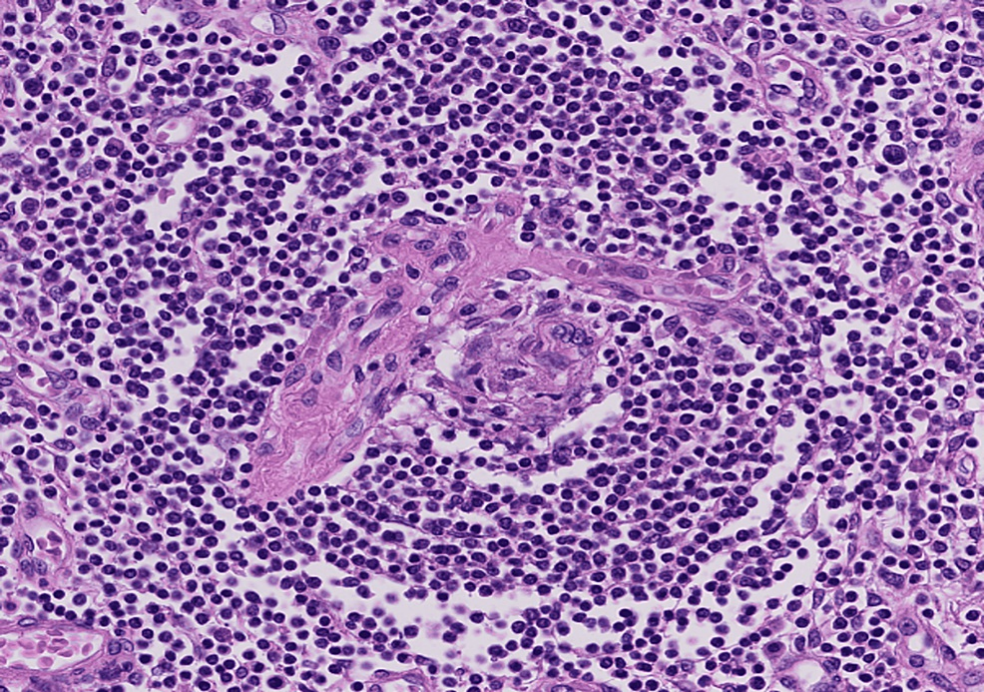
Lymph node from the tracheal bifurcation showing features of the hyaline vascular type of Castleman’s disease. Hematoxylin-eosin staining; original magnification 200×.

Furthermore, some of the organs showed features characteristic of POEMS syndrome. First, a lymph node specimen obtained at the tracheal bifurcation showed features suggestive of the hyaline vascular type of Castleman’s disease, which is known to be associated with POEMS syndrome (Figure 7).

**Figure 8:**
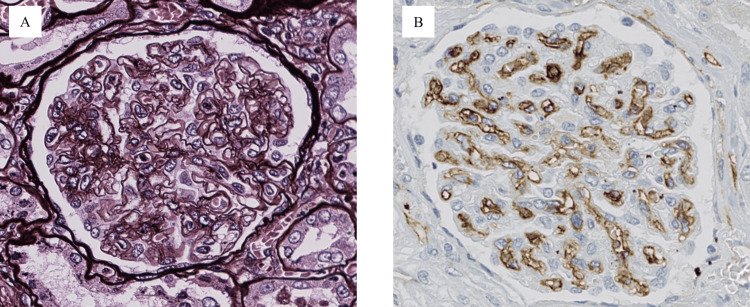
Glomerular lesions. (A) Mesangial matrix expansion and proliferation of glomerular capillary loops with the double contour of the glomerular basement membrane (periodic acid-methenamine-silver staining; original magnification 400×). (B) Glomerular basement membrane stained with anti-CD34 antibody (CD34 staining; original magnification 400×).

Second, a tissue specimen from the kidney showed mesangial matrix expansion and proliferation of glomerular capillary loops with the double contour of the glomerular basement membrane (Figure 8A). Positive anti-CD34 antibody staining indicated the proliferation of vascular endothelial cells (Figure 8B).

**Figure 9:**
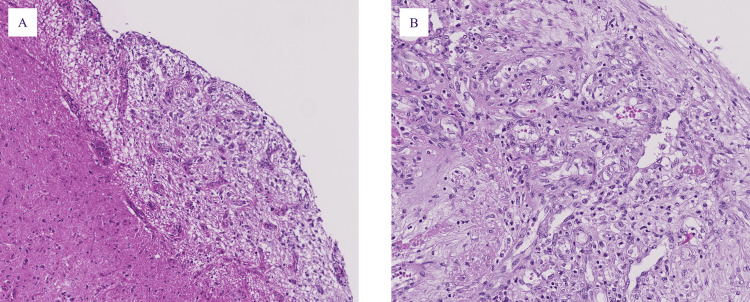
Lesions with the proliferation of microvessels. (A) Cervical cord (hematoxylin-eosin staining; original magnification 40×). (B) Endocardium of the right atrium (hematoxylin-eosin staining; original magnification 10×).

Third, there were small lesions with abundant microvessels on the surface of the cervical cord and in the right atrium and ventricle (Figures 9A, 9B).

## Discussion

This report describes a patient with POEMS syndrome who was found to have a proliferation of monoclonal IgA-lambda-restricted plasma cells in his bone marrow, although both serum SPEP and IFE suggested IgA-kappa and IgA-lambda biclonal gammopathy on a background of polyclonal gammopathy. Given that biclonal gammopathy is an atypical clinical manifestation of POEMS syndrome, making the correct diagnosis was challenging. There are several plausible explanations for the discrepancy in clonality between serum and bone marrow in our patient.

First, the inflammatory process may have masked monoclonal gammopathy. Inflammation is one of the main reasons for increased polyclonal immunoglobulin and is also evident in POEMS syndrome because of the overproduction of pro-inflammatory cytokines, such as IL-1 beta, TNF-α, and IL-6 [14]. In our case, the laboratory data confirmed that neither IgG nor IgM was restricted and that serum TNF-α and IL-6 levels were elevated, which is consistent with systemic inflammation. Furthermore, bone marrow examination revealed a clustering proliferation of lambda monoclonal plasma cells on a background of diffusely proliferating kappa plasma cells. Considering that no proliferation of monoclonal IgA-kappa plasma cells was found at autopsy, the finding of IgA-kappa proliferation on protein electrophoresis and IFE could be explained by the proliferation of polyclonal plasma cells.

Second, renal dysfunction may also be related to biclonality in serum. The lambda light chain is normally dimeric and, compared with the kappa light chain, is excreted in only small amounts by the kidney, leading to an FLCR of approximately 0.58 [12]. However, renal dysfunction disrupts elimination of not only the lambda light chain but also the kappa light chain. A majority of patients with POEMS syndrome who have high FLC-lambda and normal FLCR in serum show renal dysfunction (24 of 31 in one study [10]), which is compatible with our case.

Finally, it is unknown whether the subtype of immunoglobulin heavy chain influences clonality in serum. The findings in our case are not consistent with those in a previous report that showed IgA-secreting cases to have a higher kappa FLC than cases with the proliferation of lambda-restricted plasma cells [10]. Furthermore, in our patient, the UPEP and IFE results were compatible with a decreased renal clearance of the lambda light chain. A combination of the polyclonal proliferation of plasma cells and renal dysfunction may have led to biclonal gammopathy accompanied by a higher lambda FLC and normal FLCR in our IgA-secreting case.

To date, five cases of POEMS syndrome with serum biclonality gammopathy have been reported. However, the proliferation of biclonal plasma cells in bone marrow was confirmed in only one patient (case 3 in Table 2) [5-9]. Case 1 was found to have a combination of IgG-kappa and IgA-lambda by SPEP and was diagnosed to have dystrophic plasmacytosis with IgA-lambda but not IgG-kappa [6]. Cases 2, 4, and 5 showed biclonal gammopathy, but no details on the histopathology of the bone marrow were reported. Case 3 showed concentrations of two IgA-kappa M-proteins in serum on IFE, and immunohistochemical examination of bone marrow revealed a restricted kappa population [8].

**Table 2:** Clinical findings in the reported cases of POEMS syndrome with biclonal gammopathy. Ig: immunoglobulin: NA: not assessed

Case	Reference	Age, years	Sex	Urine	Blood	Bone marrow	Other lesions
1	[6]	58	NA	NA	IgA-lambda, IgG-kappa	NA	Biopsy of an osteosclerotic lesion and the sacrum, ilium, and acetabulum revealed dystrophic plasmacytosis with IgA immunoreactivity
2	[9]	62	Female	Bence-Jones protein	IgA-lambda, IgG-lambda	Nonspecific findings	Sacrum, ilium (nonspecific findings)
3	[8]	68	Male	NA	Two low concentrations of IgA-kappa M-proteins	A small, restricted kappa population that comprised <1% of cells and a minute population of B-cells that were not plasma cells	None
4	[7]	58	Male	Nonspecific findings	IgG-lambda, IgA-kappa	Nonspecific findings	NA
5	[5]	60	Female	NA	IgG-kappa, IgA-lambda	Proliferation of plasma cells (8%)	Pelvis
Our patient		76	Male	IgA-kappa	IgA-kappa, IgA-lambda	Proliferation of lambda-restricted monoclonal plasma cells (13%)	There were no other lesions

In terms of the prognosis, case 1 was treated with all-trans-retinoic acid followed by irradiation and survived for at least 200 days despite high levels of pro-inflammatory cytokines, including TNF-α and IL-6. The other reports did not mention the timing of diagnosis or the prognosis. Our patient died very soon after the diagnosis of POEMS syndrome. However, it is not clear whether biclonality itself contributed to the poor outcome in this patient. Patient age, extravascular volume overload, and severe renal dysfunction have been reported to affect the prognosis in cases of POEMS syndrome [1,15,16].

The main limitation of this research is that we were unable to conclude whether the biclonality seen in serum is a hallmark of a poor clinical outcome because so few cases have been reported. Furthermore, the delay in accurate diagnosis could have resulted in disease progression and a poor outcome.

## Conclusions

Biclonal gammopathy is likely to be found as a result of an inflammatory process and renal dysfunction and may mask M-protein, which is a major criterion for POEMS syndrome, even in patients in whom proliferating plasma cells secrete monoclonal immunoglobulin. This phenomenon makes precise and early diagnosis of POEMS syndrome difficult and may result in a poor outcome. Clinicians should include POEMS syndrome in the differential diagnosis if the clinical findings are compatible even if monoclonality is not obvious in peripheral samples.
